# Lentiviral Vector Purification Using Genetically Encoded Biotin Mimic in Packaging Cell

**DOI:** 10.1016/j.omtm.2018.10.008

**Published:** 2018-10-23

**Authors:** Leila Mekkaoui, Farhaan Parekh, Ekaterini Kotsopoulou, David Darling, Glenda Dickson, Gordon W. Cheung, Lucas Chan, Kirsty MacLellan-Gibson, Giada Mattiuzzo, Farzin Farzaneh, Yasuhiro. Takeuchi, Martin Pule

**Affiliations:** 1 UCL Cancer Institute, University College London, Paul O’Gorman Building, 72 Huntley Street, London WC1E 6BT, UK; 2 Autolus Limited, Forest House, 58 Wood Lane, London W12 7RZ, UK; 3 School of Cancer & Pharmaceutical Sciences, King’s College London, Molecular Medicine Group, The Rayne Institute, 123 Coldharbour Lane, London SE5 9NU, UK; 4 National Institute for Biological Standards and Control-MHRA, Blanche Lane, South Mimms, Potters Bar, Hertfordshire EN6 3QC, UK; 5 Division of Infection and Immunity, University College London, Rayne Building, 5 University Street, London WC1E 6JF, UK

**Keywords:** lentiviral vectors, synthetic peptide, biotin mimic, streptavidin, purification, affinity chromatography, competitive elution

## Abstract

Lentiviral vectors (LVs) have recently witnessed an increasing demand in research and clinical applications. Their current purification processes represent the main bottleneck in their widespread use, as the methods used are cumbersome and yield low recoveries. We aimed to develop a one-step method to specifically purify LVs, with high yields and reduced levels of impurities, using the biotin-streptavidin system. Herein, packaging HEK293T cells were genetically engineered with a cyclical biotin-mimicking peptide displayed on a CD8α stalk, termed cTag8. LVs were modified with cTag8 by its passive incorporation onto viral surfaces during budding, without viral protein engineering or hindrance on infectivity. Expression of cTag8 on LVs allowed complete capture of infectious particles by streptavidin magnetic beads. As cTag8 binds streptavidin in the nanomolar range, the addition of micromolar concentrations of biotin resulted in the release of captured LVs by competitive elution, with overall yields of ≥60%. Analysis of eluted LVs revealed high purity with a >3-log and 2-log reduction in DNA contamination and host cell proteins, respectively. This one-step purification was also tested for scalable vector processing using monolith affinity chromatography, with an encouraging preliminary overall yield of 20%. This method will be of valuable use for both research and clinical applications of LVs.

## Introduction

Over the past decade, lentiviral vectors (LVs) have become increasingly utilized in clinical gene therapy with a concomitant increased need for large-scale high specification manufacture. A major obstacle in LV manufacturing is the lack of commercially viable, robust, and scalable downstream processing that is compliant with good manufacturing practice (GMP) standards and results in high recovery and purity of final vector product. Here, we explore a single-step affinity-column viral purification method that yields high recovery with reduced levels of impurities.

Current large-scale processing schemes of LVs use multiple processes to capture viral particles, eliminate contaminants, and polish vector products.^1^, ^2^ Post-clarification, concentration is commonly achieved by ultrafiltration (UF)/diafiltration (DF) of the LV product, with tangential flow filtration being the most commonly used process.^3^, ^4^, ^5^, ^6^ Purification is then usually achieved by chromatographic means, with anion exchange chromatography (AEX) being the most commonly used in both research and clinical-grade LV applications.^7^, ^8^, ^9^, ^10^, ^11^, ^12^ As AEX results in the co-purification of nucleic acids, which currently represents the major contaminant of LV supernatant,^13^ most large-scale downstream processing schemes also include a nuclease treatment step using Benzonase, which in turn needs to be removed from the final product.^5^, ^14^, ^15^, ^16^, ^17^ Size exclusion chromatography (SEC), which suffers from product dilution and slow flow rates, is then used as a polishing step.^18^

The expression of a peptide tag on the surface of viral particles would enable the selective capture of vector particles by affinity chromatographic purification. Unlike most methods currently used, the high specificity of affinity-based purification should enable the selective capture of target vectors from virus-containing medium, which provides an increased product yield, reduce the number of steps required for purification and in turn reduce the cost of manufacturing. The expression of a tag on viral particles has typically been achieved by engineering viral envelope glycoproteins, often at the cost of loss of particle infectivity.^19^, ^20^, ^21^, ^22^, ^23^, ^24^ A few studies have reported the affinity purification of engineered LVs using ligands such as hexahistidine.^25^, ^26^ However desorption of vectors from captured columns required the use of imidazole, which has been shown to cause LV inactivation.^18^, ^24^ Therefore, although affinity chromatography is an attractive concept for LV purification, its main challenge remains the lack of a means for gentle desorption of vectors to minimize vector inactivation and retain maximum levels of infectivity.

Among the different affinity-based purifications, the biotin-(strept)avidin system, with its high affinity (dissociation constant, K_D_, ∼10^−15^ M),^27^ is one of the most utilized. The strength of this interaction however hinders the desorption and recycling of streptavidin matrices. Peptides, termed “biotin mimics” that bind streptavidin have been described. These have two advantages: first, they can be genetically encoded; and second, they bind streptavidin with a lower affinity potentially allowing the release of captured targets with biotin. Several linear streptavidin-binding peptides now exist, with affinities ranging from micromolar^28^, ^29^, ^30^, ^31^, ^32^ to nanomolar,^33^, ^34^, ^35^ and are used in various applications for research and commercial purposes. There is reason to suggest however that other structural conformations, such as cyclical biotin mimics, might be superior to linear peptides in terms of stable binding confirmation^36^ and increased ligand affinity.^37^, ^38^

Here, we expressed cyclical biotin mimetope on the surface of LV packaging cells as a type I transmembrane protein. We showed that the surface expressing mimic was passively incorporated onto LV particles during virion budding without loss of titer. This allowed LV particles to be captured on streptavidin. We then exploited the lower affinity for streptavidin of the mimetope than biotin to competitively release viral particles in physiological conditions. This system was explored as an LV purification strategy.

## Results

### Comparison of Three Biotin Mimics for Streptavidin Binding on Different Structural Formats

**Figure 1:**
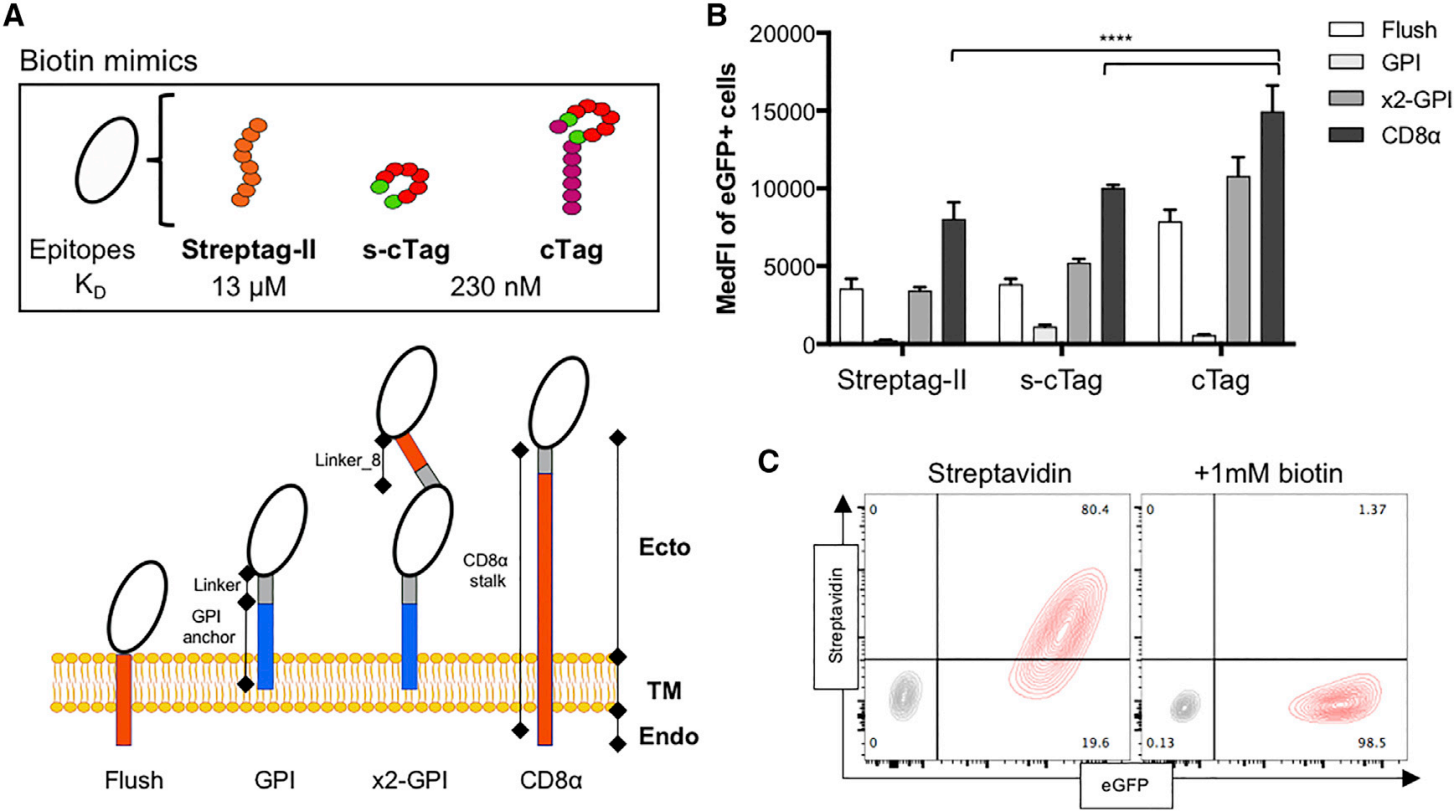
Reversible Streptavidin Binding of Synthetic Surface Expressing Biotin Mimics (A) Schematic diagrams of the chosen mimics with their reported dissociation constants (K_D_). Four surface expression structures were cloned, and their architectures are represented: (1) Flush, consisting of a CD8-derived transmembrane (TM) and endodomain (Endo); (2) GPI, consisting of GPI anchor sequence (25 amino acids [aa]), which leads to the addition of GPI at the anchor sequence, with a serine-glycine linker (6 aa); (3) x2-GPI, consisting of 2 copies of the epitopes’ open reading frame (ORF) separated by a serine-glycine linker with the first 14 aa of the CD8α stalk ectodomain (Ecto; Linker_CD8), on a GPI anchor sequence; (4) CD8α, consisting of the CD8α stalk comprising the ecto-, transmembrane, and endodomains of the human CD8α molecule, with a serine-glycine linker. All peptides were cloned into a retroviral plasmid termed “SFG,” derived from the Moloney murine leukemia virus (MoMLV), upstream of the EGFP marker gene expressed by an internal ribosome entry site (IRES). (B) 293T cells were transiently transfected with all cloned constructs and stained with APC-conjugated streptavidin 48 hr later. The median fluorescence intensity (MedFI) of streptavidin binding of EGFP-positive cells are presented in a graph indicating ±SD of three independent transient expression experiments; ****p < 0.0001. (C) Negative control 293T (gray population) and cTag8-expressing 293T cells co-expressing EGFP (cTag8 293T, red population) were first stained with streptavidin conjugated to APC, and samples were analyzed by flow cytometry. Samples were then washed and incubated with 1 mM biotin for 1 hr at room temperature and analyzed by flow cytometry. Results are presented as overlaid contour plots before (Streptavidin) and after (1 mM biotin) biotin addition.

We first sought to determine an optimal cell-membrane-expressed, genetically encodable, biotin-mimicking protein. Such a protein requires the biotin mimic to be fused to a membrane-bound spacer domain. We compared three biotin mimetopes: the linear peptide Streptag-II, (which binds streptavidin with affinity 13 μM)^32^ and two variants of a cyclical peptide, short cTag (s-cTag) and flanked cTag (CHPQGPPC and ECHPQGPPCIEGRK, respectively, which bind streptavidin at 230 nM).^39^ These mimics were fused either directly to the stalk-transmembrane domain of CD8α or to a glycosylphosphatidylinositol (GPI) anchor. Preliminary data indicated poor accessibility to the GPI anchor, so a second variant was made with two copies of each mimic (Figure 1A). Fusion proteins were co-expressed with EGFP and transfected into 293T cells, and streptavidin allophycocyanin (APC) binding was normalized to EGFP. Flanked cTag bound streptavidin with the highest median fluorescence intensity (MedFI) (14,956 ± 1,668), compared to short cTag (10,043 ± 185) and Streptag-II (8,049 ± 1,064) on CD8α stalks (Figure 1B). Both GPI variants for all mimics resulted in significantly lower MedFIs (between 3,440 ± 225 and 10,804 ± 1,204), compared to the CD8α variants (Figure 1B). As the objective was high surface expression level, the construct of the flanked cTag on a CD8α stalk, termed cTag8, was selected for further study. To further validate cTag8 as a biotin mimic, its reversible binding to streptavidin was demonstrated by biotin’s competitive binding (Figure 1C).

### Establishment of cTag8-Modified Vector Packaging Cells

**Figure 2:**
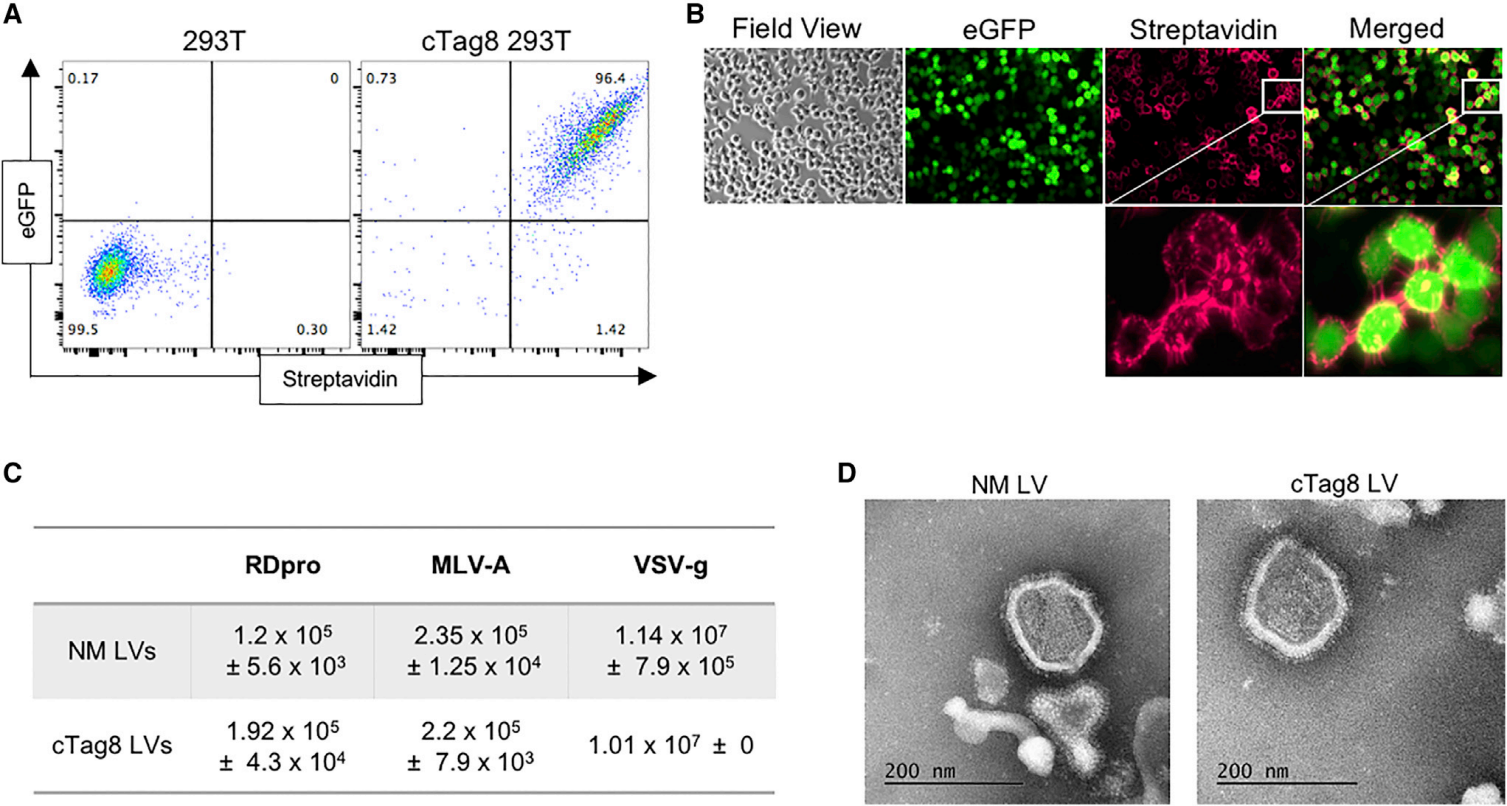
Generation of cTag8-Expressing 293T Cells for Modified Vector Production (A) Both 293T (non-transduced) and 293T cells expressing cTag8 (cTag8 293T) by γ-retroviral transduction with cTag8 co-expressed with EGFP were stained with streptavidin-APC for cTag8 expression analysis by flow cytometry. (B) Surface expression analysis of cTag8 by immunofluorescence staining of cTag8 293T cells with streptavidin-APC. Engineered cells were assessed for LV packaging capacity by the production of LVs from both 293T cells (non-modified, NM LVs) and cTag8 293T cells (cTag8 LVs) in plain DMEM, pseudotyped with either RDpro, MLV-ampho, or VSV-G glycoproteins. (C) Viral supernatants were frozen, viral titers (infectious units (IU)/mL) of stocks were determined by infectivity assay, and mean values are presented ± SD of triplicate determinations. (D) Sucrose-cushion-ultracentrifuge-purified VSV-G pseudotyped NM LVs and cTag8 LVs were negatively stained and analyzed by transmission electron microscopy (TEM). Scale bars, 200 nm.

Having shown that the mimetic protein could bind soluble streptavidin, we next sought to show that it could also bind matrix-bound streptavidin. A K562 cell line was engineered to stably express cTag8 using the same EGFP-fused construct as before, and their selection was demonstrated with streptavidin-coated paramagnetic beads (Figures S1A and S1B). With this proof of principle established, a 293T cell line stably expressing cTag8 was generated (Figures 2A and 2B) for use as a host cell line for virus production. The impact of the expression cTag8 on the ability of the 293T cell to produce LVs and any impact on subsequent lentiviral particles was then investigated. Non-modified (NM) LVs and cTag8 LVs were transiently produced from 293T and cTag8 293T cells, respectively, and transfected with a second-generation lentiviral packaging system using three different pseudotyping envelopes: RDpro,^40^ MLV-ampho, and vesicular stomatitis virus G protein (VSV-G). Viral titers were determined by infectious assay (Figure 2C). Expression of cTag8 had no effect on titer. In addition, transmission electron microscopy (TEM) of VSV-G pseudotyped NM LVs and cTag8 LVs showed no morphological differences between the virions (Figure 2D).

### Complete Capture of cTag8-Modified LVs by Passive Incorporation in Serum-free Medium

**Figure 3:**
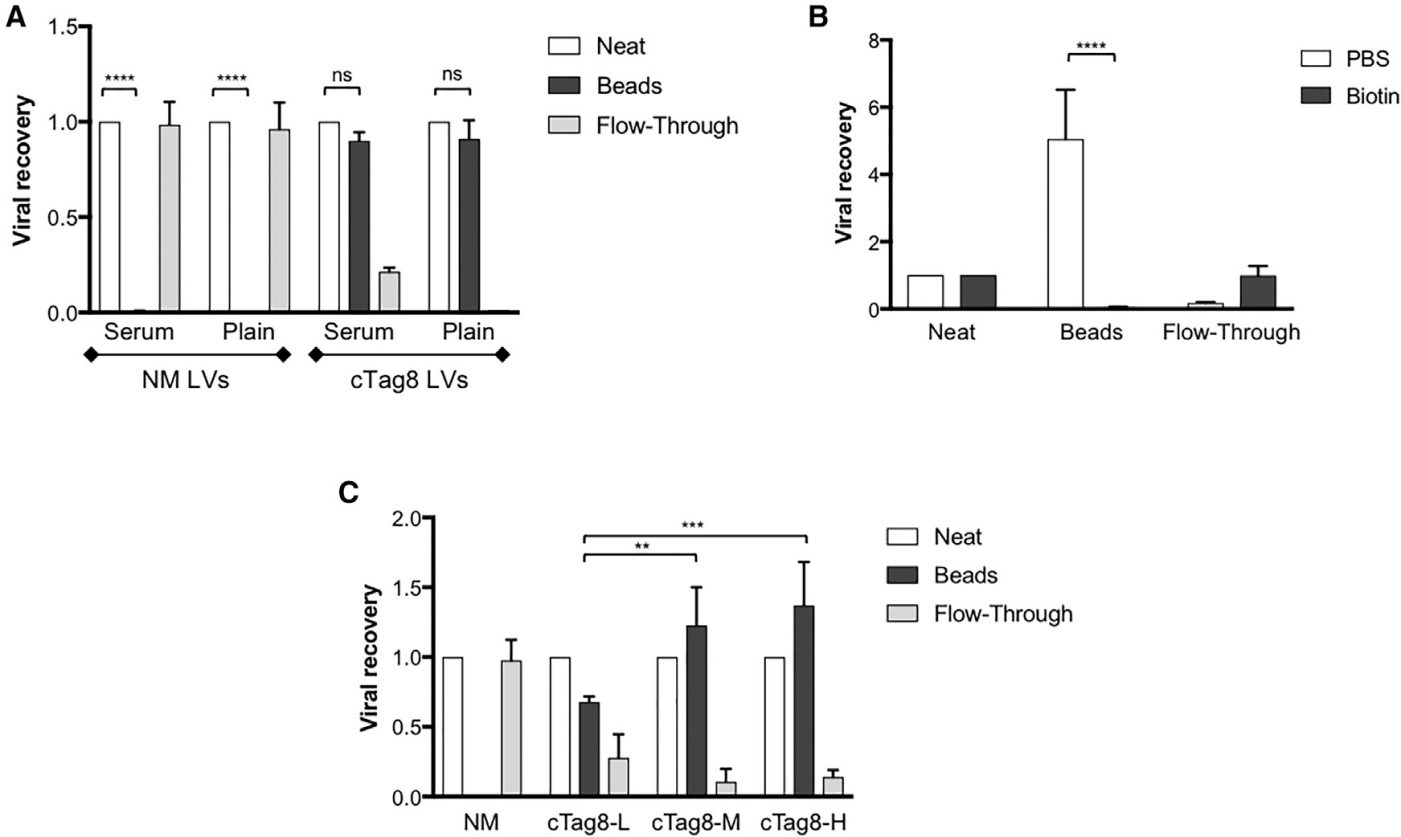
Streptavidin-Mediated Complete Capture of cTag8-Modified LVs Transiently produced LVs from 293T and cTag8 293T cells, termed NM LVs and cTag8 LVs, respectively, were incubated with streptavidin magnetic beads. Dynabeads were immobilized by magnetic capture, and flow-through fractions were collected. Streptavidin Dynabeads were then washed 4 times with cold PBS and resuspended in cold medium, in the same volume as starting viral supernatants. Viral titers (IU/mL) of all fractions: (1) crude (Neat), (2) re-suspended magnetic bead (Beads), and (3) post-capture incubation flow-through (Flow-Through) fractions were determined by infectivity assay on 293T cells. (A) NM LVs and cTag8 LVs were produced in either serum-supplemented IMDM (serum) or serum-free DMEM (plain) medium. Viral supernatants were then subjected to capture methodology for 2 hr at 4°C. (B) Streptavidin Dynabeads were pre-treated either with plain or 15 mM biotin-supplemented PBS for 1 hr at room temperature. After 3 washes with PBS, pre-treated beads were incubated with cTag8 LVs in serum-free media for 1 hr at room temperature. (C) LVs were produced in serum-free media from low (L), medium (M), and high (H) cTag8-expressing 293T cells transiently. Along with control NM LVs, all viral supernatants were incubated with streptavidin Dynabeads for 1 hr at room temperature. All values presented represent viral recovery of each fraction compared to corresponding total vector input. Data are plotted ± SD of triplicate determinations. **p ≤ 0.01; ***p ≤ 0.001; ****p ≤ 0.0001; ns, non-significant.

We next sought to determine whether viral particles produced from cTag8 293T cells could be captured on a streptavidin matrix. Since free biotin in the culture media might compete for streptavidin, consideration was given to culture media: Iscove’s modified Dulbecco’s medium (IMDM) contains 52.3 nM biotin, while DMEM contains none. Further, fetal calf serum (FCS) typically contains 2 nM biotin.^41^ Hence, 293T cells or cTag8 293T cells were cultured in either FCS-supplemented IMDM (serum) or in serum-free DMEM (plain) 24 hr after transfection with lentiviral plasmids. Harvested supernatants were 0.45 μM filtered and incubated with streptavidin Dynabeads at 0.5 mg beads per milliliter of LV supernatants for 2 hr at 4°C, with gentle rotation. Magnetic beads were then magnetically immobilized and “flow-through” fractions were collected. Filtered, but otherwise unmanipulated, supernatant (“neat” supernatant) was also kept as a control. Viral titers of neat, flow-through, and bead fractions were determined by infectious assays of 293T cells (Figure 3A). The bead fractions of both serum and plain-capture conditions resulted in no significant difference to their neat respective fractions, with 0.9 ± 0.046 and 0.91 ± 0.098 of the total viral input captured by streptavidin from cTag8 293T cells. In contrast (and as expected), no bead capture was observed with 293T cell supernatant. Further, 0.21 ± 0.021 of the cTag8 LV vector was found in the flow-through fraction in the presence of IMDM and FCS, while none was found if plain DMEM was used. Lastly, cTag8 LVs bound streptavidin in a stable manner throughout the capture process, as no infectious particles were detected in the wash fractions post-capture (Figure S2).

### Streptavidin Vector Purification Is Blocked by Biotin and Influenced by cTag8 Expression Density

To demonstrate cTag8 specificity of capture, washed streptavidin Dynabeads were incubated with either plain PBS or with PBS supplemented with an excess of 15 mM biotin for 1 hr at room temperature. After PBS washing, NM and cTag8 LVs were incubated with pre-treated beads under optimal conditions (see Figure S3 for optimization of the surface area of streptavidin beads per infectious unit and capture incubation time). After flow-through separations, infectious titers were determined for all fractions by infectivity assay (Figure 3B). Dynabeads pre-incubated with PBS only resulted in a significant 5-fold increase in the viral titer of captured cTag8 LVs, compared to starting material, with 0.17 ± 0.02 of the titer remaining in flow-through. In contrast, pre-incubation of streptavidin Dynabeads with 15 mM biotin blocked cTag8 LV binding, resulting in a lack of capture, with 0.99 ± 0.2 of cTag8 LVs found in the flow-through fraction. Accordingly, a significant statistical difference was observed between viral titers of PBS- and biotin pre-incubated Dynabead fractions with p ⩽ 0.0001, indicating that cTag8 LVs are occupying biotin-binding sites on streptavidin. Next, we tested whether there was a correlation between capture efficiency and epitope density on viral particles. To that end, cTag8 293T cells were sorted by flow cytometry into low, medium, and high cTag8-expressing cells (Figure S4). cTag8 LVs produced from these sorted populations, along with negative control NM LVs, were subjected to streptavidin purification using optimized conditions, and viral titers of all fractions were determined by infectivity assay (Figure 3C). There was a significant difference between viral titers of bead fractions and flow-through fractions produced from medium and high cTag8-expressing cells, but not low cTag8-expressing cells. These results indicated that passive incorporation of cTag8 into virions is (as expected) proportional to the density of cTag8 expression on the surface of the cells and that there is a threshold of expression, below which cTag8 incorporation onto viral particles was not sufficient for complete viral particle capture by the beads.

### cTag8-Based Purification Is Envelope Independent

**Figure 4:**
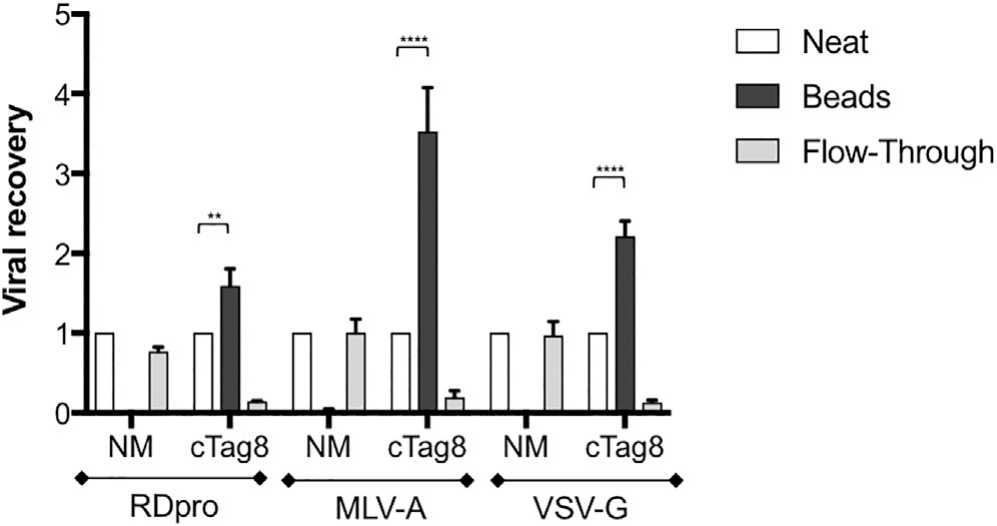
Purification of cTag8-Modified LVs in an Envelope-Independent Manner Thawed NM LVs and cTag8 LVs pseudotyped with either RDpro, MLV-ampho, or VSV-G glycoproteins were incubated with streptavidin Dynabeads for 1 hr at room temperature. Dynabeads were immobilized by magnetic capture, and flow-through fractions were collected. Streptavidin Dynabeads were then washed 4 times with cold PBS and resuspended in cold medium in the same volume as starting viral supernatants. Viral titers (international units per milliliter) of all fractions: (1) crude (Neat), (2) re-suspended magnetic beads (Beads), and (3) post-capture incubation flow-through (Flow-Through) fractions were determined by infectivity assay on 293T cells. Data represent the viral recovery of each fraction compared to corresponding total vector input and are plotted ± SD of triplicate determinations; **p ≤ 0.01; ****p ≤ 0.0001.

As this purification relies on the passive incorporation of cTag8 onto viral particles, we next aimed to demonstrate that this process can purify vectors regardless of pseudotyping envelope. For this purpose, cTag8 LVs pseudotyped with either RDpro, MLV-ampho, or VSV-G were produced from medium cTag8-expressing 293T cells. Harvested vector supernatants were incubated with streptavidin beads for 1 hr at room temperature, and infectious titers were determined for all fractions (Figure 4). cTag8-modified vectors were successfully captured by streptavidin beads, as indicated by increased viral titers of all bead fractions, with increased viral recovery of 1.60 ± 0.21, 3.53 ± 0.55, and 2.22 ± 0.19 of total viral input, compared to neat fractions, for RDpro, MLV-ampho, and VSV-G LVs, respectively (Figure 4). These results demonstrated the independence of our purification methodology from viral pseudotyping.

### Biotin Mediates Elution of Captured cTag8-Modified LVs

With a dissociation constant for cTag8-streptavidin binding in the nanomolar range, the presence of biotin should outcompete captured cTag8 LVs for streptavidin binding and, subsequently, allow the desorption of cTag8 LVs. To confirm this, optimal biotin concentration for efficient vector elution was first determined by incubating vector bound to magnetic beads with Opti-MEM containing decreasing concentrations of biotin from 15 mM to 15 fM in 100-fold serial dilutions. A concentration of 500 μM biotin was determined as optimal for subsequent experiments (Figure S5). Next, desorption efficiencies of captured cTag8 LVs were tested in the presence or absence of protein additives and in different media commonly used for virus production. Accordingly, after the capture of cTag8 LVs by streptavidin magnetic beads (Figure S6A), fractions were incubated with plain medium (X-VIVO 15, DMEM, or Opti-MEM) or supplemented with 500 μM biotin or with 500 μM biotin and 0.5% BSA. The incubation was performed for 1 hr at room temperature without any volume reduction. Eluate fractions were collected after magnetic bead immobilization, and viral recoveries were determined by infectivity assay (Figure S6B). The addition of BSA as an excipient to biotin resulted in the highest overall yields, compared to biotin only for all mediums used, with 67 ± 5.6%, 58 ± 8%, and 59 ± 9% using X-VIVO 15, DMEM, and Opti-MEM, respectively. Interestingly, in the absence of BSA, X-VIVO 15 resulted in superior elution recovery compared to DMEM and Opti-MEM, highlighting its endogenous protein composition that seemed to aid higher yields. These results indicated that our one-step purification methodology can be applied to various culturing media supplemented with biotin and BSA, resulting in ≥60% elution recoveries of infectious LVs.

### Eluted cTag8-Modified Vectors Are of High Purity

**Figure 5:**
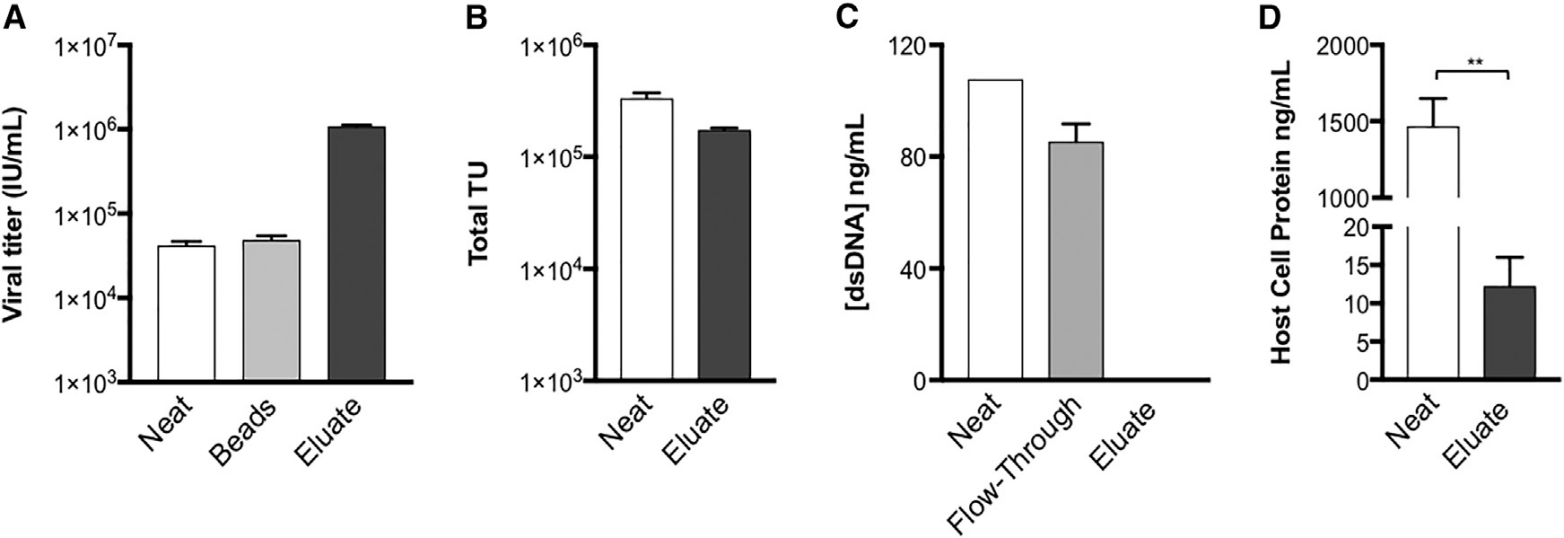
Biotin-Mediated Concentration and Purification of cTag8 LVs LVs produced from cTag8 293T cells were incubated with streptavidin Dynabeads for 1 hr at room temperature. Dynabeads were then washed 4 times with PBS and resuspended in plain DMEM and a fraction was collected. Subsequently, Dynabeads were magnetically immobilized and resuspended in Opti-MEM supplemented with 500 μM biotin and 0.5% BSA in 1/50^th^ of the starting volume and incubated for 1 hr at room temperature. Post-magnetic immobilization of Dynabeads, concentrated eluted LVs (eluate) were collected. (A) Viral titers (IU/mL) of starting (Neat), captured (Beads) and elution (Eluate) fractions were determined by infectivity assay, from which (B) the total transducing units (Total TU) in starting and eluted fractions were calculated. (C) Concentrations of dsDNA were quantified in collected viral fractions by PicoGreen analysis (detection limit < 1 ng/mL). (D) Total immunoreactive HEK293 host cell proteins (HCPs) were detected in starting and eluted LV fractions. All values are presented ± SD of three independent experiments; **p ≤ 0.01.

High vector purity is as important as infectious particle recovery and a prerequisite for an effective downstream purification vector process. To demonstrate the efficiency of our purification, captured cTag8 LVs were eluted in 50× reduced volume (compared to that of the starting supernatant material) using Opti-MEM supplemented with 500 μM biotin and 0.5% BSA (Figure 4A). Eluted viral vectors resulted in a 26-fold increase in viral titer (1.09 ± 0.04 × 10^6^ IU/mL), which represented 60% of the starting viral particles (Figures 5A and 5B). Subsequently, to determine the purity achieved with this method, eluted vectors were analyzed for process-related impurities. First, double-stranded DNA (dsDNA) concentrations were determined with the PicoGreen assay kit, which can detect down to 1 ng/mL nucleic acids. As dsDNA does not specifically bind to streptavidin, the majority of dsDNA from starting material (108 ng/mL) was detected in the flow-through fractions, whereas undetectable levels of dsDNA were found in the concentrated eluate (Figure 5C). Taking the total volume of both starting material and eluted LVs, our one-step purification led to the removal of >99.98% (>3-log) of the total dsDNA. Second, host-cell protein (HCP) contamination was assessed in biotin-displaced cTag8 LVs. HEK293 host cell proteins were quantified in both starting supernatant and purified vectors using an HEK293 HCP ELISA kit (Figure 5D). 1,468 ± 181 ng/mL HCP was detected in starting neat supernatant, compared to 12 ± 4 ng/mL detected in purified cTag8 LV eluate, eliminating 99.8 ± 0.04% of the total HCP (p = 0.0051). Therefore, our developed affinity purification resulted in a 2-log reduction of host cell DNA and protein impurities in a one-step viral purification process.

### Scalability of Purification Using Streptavidin-Based Affinity Chromatography

**Figure 6:**
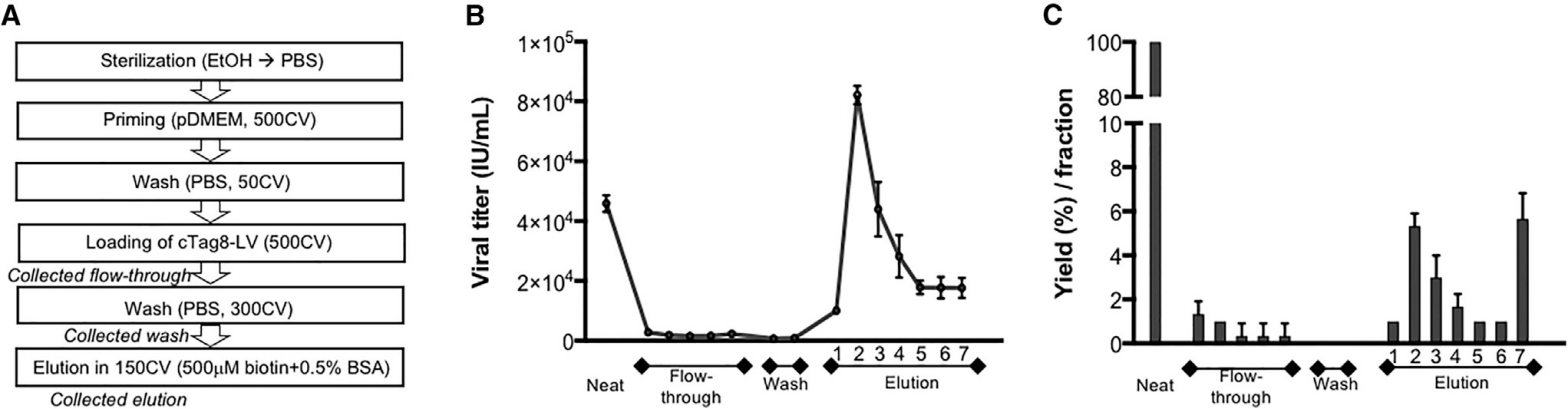
Affinity Chromatography Purification of cTag8 LVs Using Streptavidin Monolith Column (A) Diagram of the CIMac streptavidin (0.1-mL) analytical column purification step process, with indicated collected fractions (5 × 10 mL flow-through fractions; 2 × 15 mL wash fractions; 6 × 1.5 mL [1 to 6], and 1 × 7.5 mL [7] eluate fractions), which were assessed for their viral titers by infectivity assay. (B) Viral titers of collected fractions, and (C) the yield of each fraction in terms of total transducing units (TU), which are represented as a percentage of starting input transducing units. Values are presented ± SD of triplicate determinations for each fraction.

To determine the suitability of this purification to process large volumes of cTag8 LVs, we next tested viral capture in monolith-based columns. We selected CIMac high-performance streptavidin columns (BIA Separations). As this type of column has never been tested for chromatographic purification of LVs, a preliminary strategy using a column with a column volume (CV) of 0.1 mL and streptavidin density of 2 μg/mm^2^ was established to attempt to capture and elute cTag8 LVs by affinity chromatography (Figure 6A). Subsequent to priming the column, 500 CV of serum-free RDpro-pseudotyped cTag8 LV was loaded onto the column, and flow-through was collected in 10-mL fractions. Next, 150 CV of X-VIVO 15-based elution buffer containing a saturating concentration of 15 mM biotin, supplemented with 0.5% BSA, was loaded into the column; and six elution fractions of 15 CV (i.e., 1.5 mL) (E1–E6) were collected, followed by a final 75 CV fraction (E7). All collected fractions were then assayed for viral titer by an infectivity assay (Figure 6B). The loading of cTag8 LVs supernatant (4.58 ± 0.28 × 10^4^ IU/mL, total yield 2.29 x10^6^ TU) onto the column resulted in good vector binding to immobilized streptavidin, as indicated by the presence of only 9.11 × 10^4^ total transducing units (TU) detected in all of the five 10 mL collected flow-through fractions, which represented 4% of total TU (Figure 6C). Moreover, washing the column did not displace any bound LVs, indicating specific and high binding affinity of cTag8 LVs to the column. Furthermore, the loading of the elution buffer onto the column resulted in the gradual desorption of cTag8 LVs from the column, with a clear eluted viral peak in E2 containing a concentrated titer of 8.21 ± 0.3 × 10^4^ IU/mL, representing an 1.8- ± 0.11-fold increase compared to starting vector material. The elution of cTag8 LVs continued in E3 with 4.40 ± 0.91 × 10^4^ IU/mL and in E4 with 2.82 ± 0.71 × 10^4^ IU/mL, which represented 96% and 61% of the starting titer, respectively. Interestingly, in the last collected fractions of E5 to E7, cTag8 LV displacement continued in a stable manner, with similar titers in all three fractions of 1.77 × 10^4^ IU/mL, representing 38% of the input titer. Taking into consideration the volume of each fraction, an overall yield of 20% was achieved in all elution fractions by biotin displacement of cTag8 LVs in this preliminary affinity chromatography run.

## Discussion

Here, we report a novel, one-step LV purification methodology achieved by the passive incorporation of a genetically encoded cyclical biotin mimic onto viral particles, which allowed the capture of LVs with reversible binding by biotin outcompetition, for both small- and large-scale applications. To the best of our knowledge, this report represents the first use of a synthetic biotin-mimicking peptide for the purification of LVs.

The current downstream processing technologies for LV purification represent a major bottleneck for the advancement of these vectors in both pre-clinical and clinical applications. The inefficiency of currently used methods is emphasized by the requirement of purification schemes, which consist of at least 4 different steps or technologies.^5^, ^14^, ^15^, ^16^, ^17^ These methods aim to either debulk the surrounding supernatant from viral vectors (e.g., UF/DF and SEC) or rely on the overall charge of viral particles for purification (e.g., AEX). Although AEX suffers from low specificity and co-elution of negatively charged impurities, it is currently the most widely applied purification technology for both laboratory and clinical scale LV purification, with several commercial kits available, such as Merck Millipore’s Fast-Trap kit, ABM’s PuRetro LV kit, Cell Biolabs’s ViraBind LV kit, and Sartorius’s Vivapure LentiSELECT kit. However, desorption from AEX matrices requires the use of harsh reagents; thus, efficient elution comes at the cost of the most important objective of any downstream process, which is to maintain vector infectivity. Thus, all of these limitations and the constraint in utilizing at least 4 technologies in currently applied multi-step schemes are, in turn, translated into the currently acceptable overall yield of 30% for clinical downstream processing of LVs, with published yields ranging between 20% and 40%.^2^, ^42^

It is well recognized that the development of a specific purification strategy based on affinity chromatography holds promising potential to increase overall yield and in turn decrease the total cost of goods in LV manufacturing, as fewer steps would be required. However, to date affinity purification of LVs has had limited success, as vector specificity has not yet been coupled with gentle desorption from affinity ligands. These two features are key in any viral purification method for its efficacy in vector manufacturing, both technically and economically. Thus, the development of a cost-effective affinity purification with gentle elution conditions, such as competitive elution, is highly desirable for the widespread use of LVs in both clinical and research applications. Accordingly, this work has addressed current limitations in LV downstream processes by the establishment of a novel one-step specific vector purification, which selectively isolated passively modified LVs followed by gentle desorption, resulting in competitive overall yields with remarkably high levels of product purity.

A precedent to this project was the metabolic desthiobiotinylation of LVs with their subsequent purification using monomeric avidin.^43^ As desthiobiotin binds avidin at a lower affinity than biotin,^44^ the loading of 2 mM biotin onto the columns with adsorbed desthiobiotinylated vectors resulted in the high recovery of 68% of the infectious virus. However, this strategy has several limitations due to the relative complexity of viral engineering, involving the co-expression of three exogenous proteins in packaging cells. Moreover, the synthesis of desthiobiotin in these cells requires the addition of 7-DAPA into the culture, which may contain traces of biotin, rendering this strategy ineffective for complete viral capture.

In our strategy, the cell surface expressing synthetic biotin mimic, cTag8, was genetically engineered into packaging cells. This circumvents the need for metabolic conjugation while having the same advantage as desthiobiotin allowing facile displacement with biotin. It should also be noted that the nature of our vector modification would also incorporate the mimic onto non-virus-cell-derived vehicles produced from cTag8-expressing packaging cells, such as exosomes, which in turn would lead to their co-purification along with viral vectors. Nevertheless, as these particles share both size and charge to LVs, their co-purification using current technology for LV purification is common. Interestingly, the developed purification presented in this study may be of use for non-virus-cell-derived particle purification.

The specificity of this affinity-based isolation was shown to be independent of viral envelope glycoproteins, demonstrating general viral vector applications. Following the complete capture of cTag8-modified LVs, biotin’s competitive binding to streptavidin routinely resulted in overall yields of ≥60% from magnetic beads and 20% from the preliminary chromatographic monolith-based purification. Greater recovery yields from the affinity chromatography could be achieved by further optimization of column desorption conditions. Optimal elution recovery was achieved by the addition of BSA as an excipient in the elution buffer. However, recombinant human serum albumin could be used as a substitute, which does not require characterization and removal from the final vector product if intended for clinical application.

Furthermore, the selectivity of our purification was demonstrated with the characterization of eluted LVs in terms of process-related impurities. The selective capture of cTag8-expressing viral particles followed by their elution in a biotin-dependent manner resulted in a >3-log and a 2-log reduction in dsDNA (<1 ng/mL final concentration) and host-cell-derived protein (12 ng/mL final concentration) contaminants, respectively. These reductions are comparable and competitive to reported values for both impurities in final LV product after its subjection to the multi-step purification schemes used, which in striking contrast, include benzonase treatments as well as at least one chromatographic purification step. Thus, this methodology seems to result in superior impurity removal in a one-step process compared to currently applied multi-step schemes.

The integration of such a vector-specific purification technique, which combines high yield with high purity, would be highly advantageous for the clinical manufacturing of LVs. The reduction of current schemes, after vector clarification, to potentially just one affinity-based chromatographic purification step would simultaneously decrease the cost of processing and increase volume bioprocessing, thus releasing the current bottleneck in LV manufacturing. The indirect nature of cTag8 modification on viral particles effectively demonstrates that our developed purification may represent a universal isolation technique for viral vectors produced either transiently or from a stable packaging or producer cell line.

## Materials and Methods

### Cell Culture

HEK293T/17 (ATCC, CRL-11268) cell line was cultured in IMDM (Lonza) supplemented with 10% FCS (Biosera, FB 1001/500) and 2 mM GlutaMAX (GIBCO) at 37°C with 5% CO_2_. 293T cells were passaged 1:4–1:10 when cell density reached 75%–85% confluence using trypsin-EDTA solution (Sigma).

### Viral Vector Production

Viral vector production was achieved by transiently transfecting 293T cells in 100-mm plates using GeneJuice (Merck Millipore, 70967), with a total of 12.5 μg DNA. γ-retroviral vectors were produced by triple transient transfection of 4.69 μg Peq-Pam plasmid (encoding Moloney GagPol), 3.13 μg RDF plasmid (encoding RD114 envelope), and 4.69 μg retroviral backbone SFG^45^ expressing the gene of interest. Supernatants were collected 48 hr and 72 hr post-transfection and frozen at −80°C. LVs were generated by GeneJuice transfection of cells with 5.42 μg pCMV-dR8.74 (encoding lentiviral GagPol), 2.92 μg envelope plasmid (pMD2.G, RDpro,^40^ or MLV-ampho-expressing plasmid, gifts from Dr. Yasu Takeuchi, University College London), and 4.17 μg lentiviral backbone pCCL encoding the gene of interest. Supernatants were collected 48 hr after transfection and processed by centrifugation at 1,000 × *g* for 10 min at 4°C to remove cellular debris, followed by ultrafiltration using Minisart NML 0.45-μm filters (Sartorius). Viral supernatants were either kept on ice for 2 hr for further use or frozen at −80°C for storage.

### Retroviral Modification of 293T Cells

Experiments were performed in 6-well plates (250,000 cells per well). Culture media were replaced the day after seeding with γ-retroviral vector supernatant carrying cTag8 co-expressed with the EGFP marker gene, in the presence of 5 μg/mL polybrene (Merck Millipore). Transduced cells (cTag8 293T cells) were harvested 72 hr later and recovered by culturing in serum-supplemented IMDM for two passages before use as lentiviral packaging cells.

### Determination of LV Titer

Functional viral titers were determined by flow cytometry analysis (using a BD LSRFortessa X-20 cell analyzer) of transgene expression in transduced 293T cells at different dilutions. Experiments were performed in 24-well plates (50,000 cells per well). Serially diluted viral supernatants (concentrated or neat un-concentrated) were added onto seeded cells in the presence of 5 μg/mL polybrene. Transduction efficiencies were determined 72 hr later, and transgene expressions between 0.5% and 20% were used in the following equation to determine viral titer.

$$\text{Titer}\;\left(\frac{\text{Infectious}\;\text{units }\left(\text{IU}\right)}{\text{mL}}\right)=\left(\frac{\left(\frac{\text{\%}\;\text{transduction}\;\text{efficiency}}{100}\right)\times \text{no}.\;\text{of}\;\text{cell}\;\text{at}\;\text{transduction}}{\text{vector}\;\text{volume}}\right)\times \text{dilution}\;\text{factor}$$

### Biotin-Mimetope-Mediated Purification of Modified LVs Using Streptavidin Dynabeads

LVs produced from both 293T and cTag8 293T cells were either left in supplemented IMDM or gently washed with PBS 24 hr post-transfection and cultured in plain DMEM. Viral supernatants were harvested 24 hr later, and after processing, fresh, frozen, or thawed crude supernatants were incubated with Dynabeads MyOne Streptavidin T1 (Thermo Fisher, 65601) magnetic beads (0.5 mg beads per milliliter of LV supernatants) that were previously washed with PBS 4×, per the manufacturer’s protocol, and resuspended in 1× PBS. Capture conditions were incubated for 15 min to 2 hr at 4°C or 37°C, as stated per experiment, with gentle rotation. Tubes were placed on a magnetic rack and left to stand for 1 min, followed by the separation of the supernatant from the immobilized magnetic beads (flow-through fraction, 1× volume). Tubes were removed from the magnetic rack, and beads were gently resuspended with PBS and placed again on the magnetic rack for 1 min for bead immobilization. This washing step was repeated 3 times, for a total of 4 washes, and beads were then resuspended in supplemented IMDM or plain DMEM to collect a bead fraction (1× volume). After resuspension for fraction collection, streptavidin beads were magnetically immobilized and resuspended in one the following elution buffers (1× volume): DMEM (GIBCO), Opti-MEM (GIBCO), or X-VIVO 15 (Lonza), with biotin and/or BSA (as mentioned per experiment), both supplied from Sigma-Aldrich. Elution steps were incubated for 1 to 2 hr at 4°C or room temperature (as stated per experiment) with gentle rotation; subsequently, tubes were placed on a magnetic rack, and eluates were collected after bead immobilization.

### Determination of Streptavidin Dynabead Purification Efficiency

All fractions (neat supernatants, bead fractions, flow-throughs, and bead eluates) were titered on 293T cells by incubating fractions with cells in the presence of 5 μg/mL polybrene to determine viral titer. For the titration of the bead fractions, LVs bound to beads were incubated with cells. To avoid the presence of beads during the flow cytometry assessment of transgene expression, all transduced conditions were split 1:2–1:3 72 hr post-transduction to dilute out the magnetic beads. 48 hr later, transduction efficiencies of the EGFP transgene were determined by flow cytometry using the BD LSRFortessa X-20 cell analyzer.

### Streptavidin Analytical Monolith Column Purification of Modified LVs

Based on the manufacturer’s recommendation, the column was placed in an upright position and connected to a peristaltic pump using connector tubes. The column fitted was washed with 50 CVs of PBS with a flow rate of 1.5 mL/min. Subsequently, the column was primed with 500 CVs of plain DMEM, which is equivalent to vector-containing media of viral supernatants. After the column was washed with 50 CVs of PBS, 500 CVs (i.e., 50 mL) of serum-free RDpro-pseudotyped cTag8 LV were loaded into the system at 1.5 mL/min, and five flow-through fractions of 10 mL were collected. The column was then washed twice with 300 CV with PBS, and wash flow-throughs were also collected. Next, an isocratic elution was performed by loading 15 mL X-VIVO 15-based elution buffer, containing 15 mM biotin supplemented with 0.5% BSA, into the column, which represented a third of the cTag8 LV total loaded volume for the concentration of eluted vector. With a dead volume of 4 mL in the tubing system and the fact that X-VIVO 15 is colored compared to PBS, two fractions of 1.5 mL, termed E1 and E2, were collected, as they represented the point of mixture between wash and elution solutions. Four elution fractions of 1.5 mL were then collected (E3–E6), followed by a final 7.5-mL elution fraction (E7).

### Immunofluorescence

cTag8 293T cells were cultured in 24-well plates overnight at 5 × 10^5^ cells per well. The medium was removed, and cells were gently washed with PBS. Cells were then fixed with 4% paraformaldehyde in PBS for 20 min at room temperature and then gently washed 3 times with PBS. To block non-specific binding, PBS + 0.1% BSA was added for 30 min. Streptavidin-APC (BioLegend) was then added in blocking solution at 1:100 for 1 hr. Wells were washed 3 times with PBS after staining removal. Nuclear staining was carried out using propidium iodide (Thermo Fisher Scientific) in blocking solution at 1:3,000 for 5 min. Wells were washed 3 times with PBS and then imaged using PBS ZEISS fluorescence microscopy with the Colibri illumination system.

### Sucrose Cushion Purification

Processed LVs were gently layered on freshly prepared 20% sucrose in 50 mM sodium phosphate in ultracentrifuge tubes. Tubes were spun at 23,000 rpm for 2 hr at 4°C in a SW 32 Ti swinging-bucket rotor (Beckman Coulter). Pellets were gently resuspended in Opti-MEM and left on ice for 1 hr for optimal dissociation. Viral vectors were aliquoted and stored at −80°C.

### Electron Microscopy

Negative staining was performed by the sequential drop method; briefly, samples were adsorbed onto carbon-formvar electron microscopy (EM) grids (Agar Scientific, Stansted, UK) for 1 min; then each grid was placed sample side down on top of a 50-μL droplet of deionized H_2_O for 30 s, followed by a 2-min staining on top of a 50-μL droplet of 2% aqueous ammonium molydbate (pH 7.5) (Agar Scientific). Excess stain was wicked off using Whatman Grade 1 filter paper, and the grid was allowed to air dry. Grids were examined at 200 kV in a JEM-2100 TEM microscope (JEOL, Welwyn Garden City, UK) at 10–60,000× magnification, and areas with the most even stain levels were selected for image acquisition using a Gatan Ultrascan 4000 charge-coupled device (CCD) camera (Gatan, Pleasanton, CA, USA).

### Cell Sorting by Flow Cytometry

1 × 10^6^ cTag8 293T cells were sorted using a BD FACSAria III sorter, based on EGFP expression, into round-bottom fluorescence-activated cell sorting (FACS) tubes containing 0.5 mL 100% FCS supplemented with Normocin at 100 μg/mL (Invivogen). Samples were then moved into flasks and incubated at 37°C with 5% CO_2_. After two rounds of cell division, recovered populations were assessed for sorting efficiency by flow cytometry staining using a BD LSRFORTESSA X-20 cell analyzer.

### DNA Quantification

Double-stranded DNA contamination was assessed in purified vectors using the Quant-iT PicoGreen dsDNA Assay Kit (Invitrogen). The manufacturers’ protocol was followed using a Thermo Scientific Nunc F96 MicroWell black polystyrene plate. Absorbance was measured using the Varioskan LUX multimode microplate reader and analyzed using the SkanIt software.

### HCP Quantification

Total HCP was measured by ELISA with the HEK293 HCP Kit (Cygnus Technologies, F650). The manufacturers’ protocol was followed with diluent buffer (Cygnus, I028-500). Absorbance was measured using the Varioskan LUX multimode microplate reader and analyzed using the SkanIt software.

### Statistical Analysis

An unpaired two-tailed Student’s t test was used for comparison of matched values with only two sets of data present. A two-way ANOVA was used for comparison of groups with two variables present across more than two sets of data. Generation of graphs and statistical analysis were performed using Prism 7.0 software.

## Author Contributions

Conceptualization, L.M., E.K., Y.T., and M.P.; Methodology, L.M. and F.P.; Investigation, L.M., G.D., D.D., C.W., and K.M.-G.; Writing – Original Draft, L.M. and M.P.; Writing – Review & Editing, D.D., G.D., K.M.-G., G.M., E.K., Y.T., and M.P.; Resources, G.D. and D.D.; Funding Acquisition, M.P.

## Conflicts of Interest

The authors have no conflicts of interest.

## Supplemental Information

Document S1. Figures S1–S6

Document S2. Article plus Supplemental Information
