# Supplementary material for: Lentiviral Vector Purification Using Genetically Encoded Biotin Mimic in Packaging Cell
Source: Mol Ther Methods Clin Dev. 2018 Oct 23;11:155–65. doi: 10.1016/j.omtm.2018.10.008 (PMC6258877; doi:10.1016/j.omtm.2018.10.008)
Supplement: Document S1. Figures S1–S6 [file mmc1.pdf]

OMTM, Volume *11*

Supplemental Information

Lentiviral Vector Purification Using Genetically Encoded Biotin Mimic in Packaging Cell

Leila Mekkaoui, Farhaan Parekh, Ekaterini Kotsopoulou, David Darling, Glenda Dickson, Gordon W. Cheung, Lucas Chan, Kirsty MacLellan-Gibson, Giada Mattiuzzo, Farzin Farzaneh, Yasuhiro. Takeuchi, and Martin Pule

Supplemental Figure 1

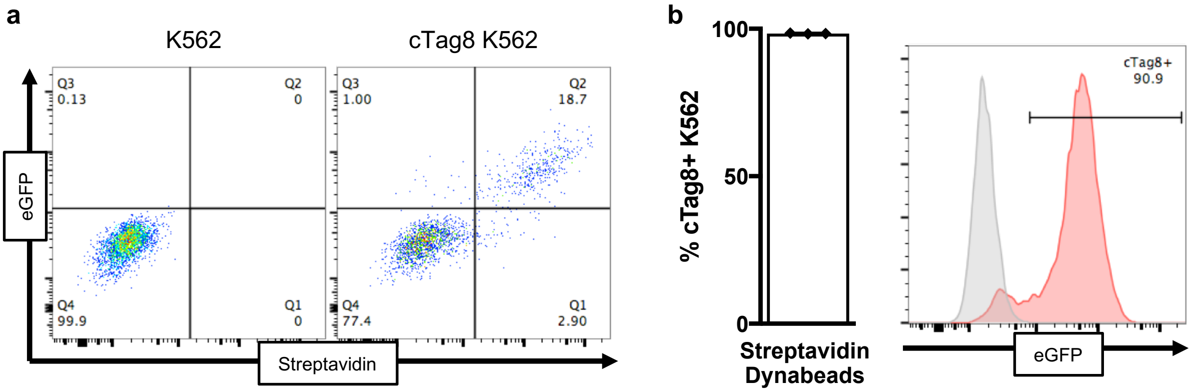

Supplemental Figure 2

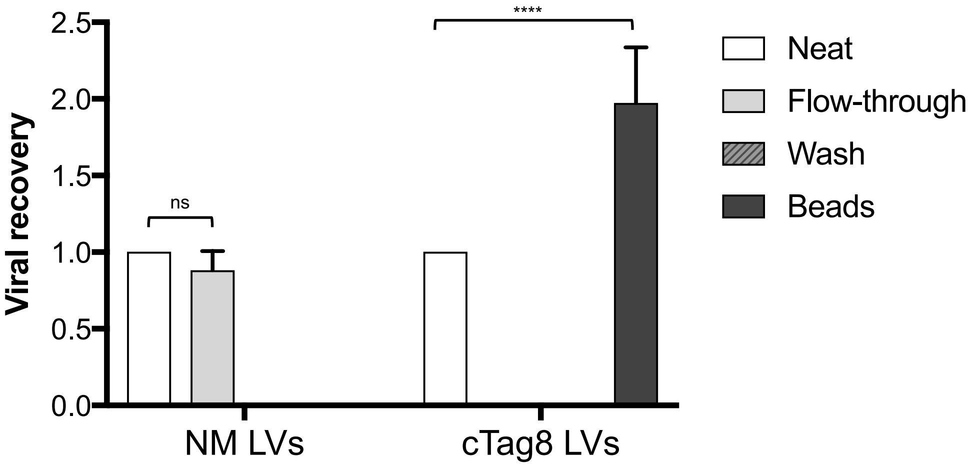

Supplemental Figure 3

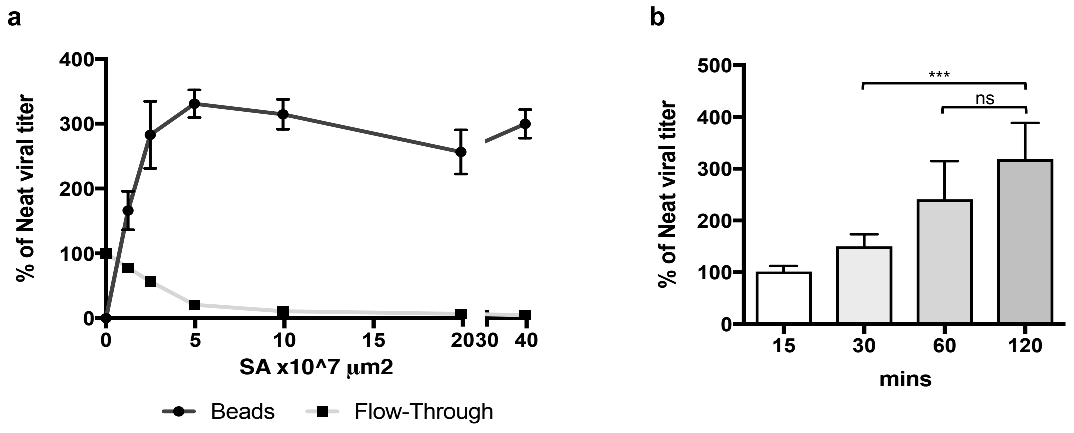

Supplemental Figure 4

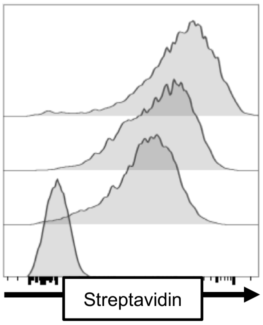

	Streptavidin MedFI of eGFP+ cells
H	14163 ± 622
M	4801 ± 459
L	3073 ± 493

Supplemental Figure 5

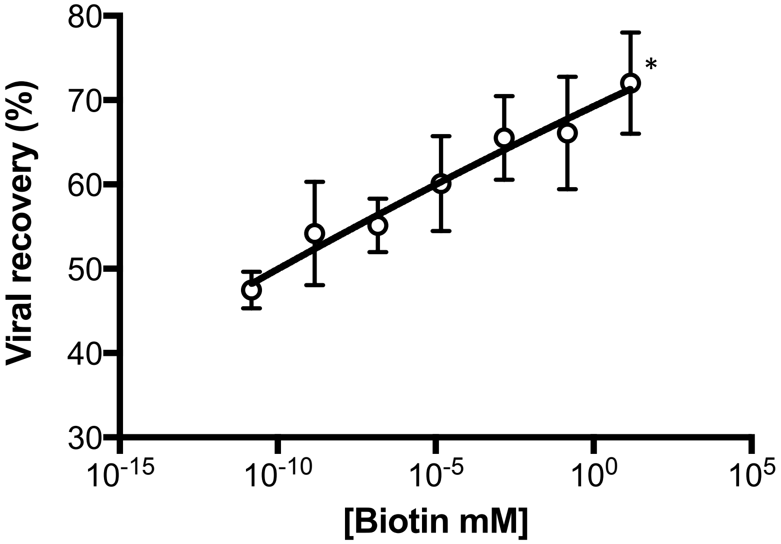

Supplemental Figure 6

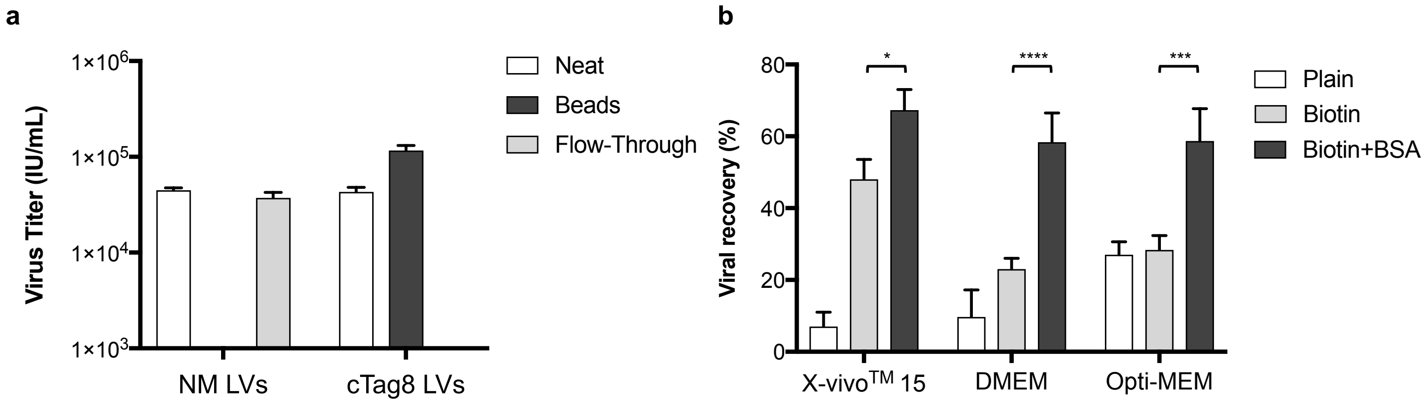

Supplemental Data

FIG. S1: Proof on concept for cellular sorting using cTag8 and streptavidin interaction.
K562 suspension cell line were γ-retrovirally transduced to express cTag8 on their surface, co-expressed with eGFP marker gene. **(a)** cTag8 expressing K562 and negative control K562 cells were stained with streptavidin-APC to determine transduction efficiency by flow cytometry; with 18.7% of the cells were double positive for streptavidin and eGFP. Transduced cells were then sorted with Dynabeads® MyOne™ Streptavidin T1 magnetic beads (10mg/mL) by incubating beads with cells for 1 hour at room temperature. Streptavidin Dynabeads were magnetically separated from the flow through and collected for subsequent cell culture. **(b)** cTag8 expression of collected Dynabeads-sorted cTag8 K562 cells was assessed 12 days post-sorting by flow cytometry. Results are presented as a percentage graph of cTag8 positive cells ± standard deviation (SD) of triplicate determinations; and an overlaid histogram of K562 (grey population) and Dynabeads-sorted cTag8 K562 (red population) as representative data.

FIG. S2: Stable cTag8 and streptavidin interaction during vector capture.
cTag8 LVs, along with negative control NM LVs, were incubation with streptavidin Dynabeads for 2h at 4°C. Beads were then immobilized by magnetization and flow-through fractions were collected. Next beads were washed 4 times with cold PBS and wash fractions were collected. Magnetic beads were then resuspended in serum-free DMEM and viral titers of all collected fractions (neat, flow-through, wash and beads) were determined by infectivity assay. Results are plotted as viral recovery of each fraction compared to total vector input ± standard deviation (SD) of three independent experiments with **** $p \leq 0.0001$ and non-significant as ns.

FIG. S3: cTag8 LVs streptavidin-mediated capture optimization.
(a) Number of magnetic beads, in terms of surface area (SA) (with 3.14 μm^2/bead and 3.16×10^5 beads/µL) per mL of LV supernatant, was optimized as follows. Beads starting with 100µL (9.92×10^7 μm^2 SA) of beads/mL of LV serial diluted 1:2 for 6 points, ending with 3.125µL (3.1×10^6 μm^2 SA) of beads/mL of LV, were washed and incubated with 1mL of cTag8 LVs (Neat viral titer of $7.6 \pm 0.4 \times 10^4$ IU/mL) for 2h at 4°C. For appropriate analysis, bead resuspension volumes after capture were kept the same as starting material. Washed virion-bound-beads and respective flow-through fractions were then assessed for infectivity. **(b)** Capture incubation time of cTag8 LVs with 25µL of beads/mL of LV was optimized by incubation reactions for 15 to 120 minutes (mins), at room temperature. Results are plotted as percentage of starting (neat) viral titer ± standard deviation (SD) of three independent experiments with *** $p \leq 0.001$ and non-significant (ns) $p > 0.05$.

FIG. S4: Establishing differential cTag8 293T cells expressors.
Populations were sorted using BD FACSARIA III sorter. Half-offset overlaid histograms of non-transduced 293T and sorted cTag8 293T cells into low (L), medium (M) and high (H) expressers stained with streptavidin conjugated to APC; with streptavidin median fluorescence intensity (MedFI) values of eGFP positive cells are presented in a table ± standard deviation (SD) of triplicate determinations.

FIG. S5: Dose-dependent biotin-mediated elution.
cTag8 LVs from vector stock were captured by streptavidin Dynabeads and bead fraction was incubated with seven 1:100 serial dilutions of biotin in Opti-MEM supplemented with 0.5% BSA, for 2h at 4°C. (*) Highest biotin concentration tested was 15mM due to the difficulty in biotin solubility at concentrations >30mM. Dose-dependent displacement graph of percentage of cTag8 LVs recovered in each elution conditions relative to starting neat supernatant ± standard deviation (SD) of three independent experiments are presented. Biotin concentrations values on the X-axis were transformed into log values for the non-linear regression analysis of the results.

FIG. S6: Efficient cTag8 LVs recovery using different culture media as elution formulation.
(a) NM LVs and cTag8 LVs were captured by streptavidin Dynabeads by incubation for 1h at room temperature. **(b)** cTag8 LV bead fraction was then subjected to elution in three different media: X-vivoT^{M}15, DMEM and Opti-MEM, each supplemented with plain, 500µM biotin and 500µM biotin with 0.5% BSA. Viral titer and recoveries of collected fractions and conditions were determined by infectivity assay. All values are represented with ± standard deviation (SD) of three independent experiments, with * $p \leq 0.05$ and *** $p \leq 0.001$.
